# Innate Immunity in Cancer Biology and Therapy

**DOI:** 10.3390/ijms241411233

**Published:** 2023-07-08

**Authors:** Yuxia Zhang, Wenjing Xue, Caili Xu, Yanyang Nan, Shuang Mei, Dianwen Ju, Shaofei Wang, Xuyao Zhang

**Affiliations:** 1 Department of Biological Medicines & Shanghai Engineering Research Center of Immunotherapeutics, School of Pharmacy, Fudan University, Shanghai 201203, China; 20301030096@fudan.edu.cn (Y.Z.); 22211030033@m.fudan.edu.cn (W.X.); 21111030069@m.fudan.edu.cn (C.X.); 20111030070@fudan.edu.cn (Y.N.); dianwenju@fudan.edu.cn (D.J.); 2 Shanghai Tinova Immune Therapeutics Co., Ltd., Shanghai 201413, China; s.mei@novatim-zj2.com; 3 Department of Cellular and Genetic Medicine, School of Basic Medical Sciences, Fudan University, Shanghai 200032, China

**Keywords:** innate immunity, cancer therapy, immune checkpoint inhibitors, chimeric antigen receptors, cytokines

## Abstract

Immunotherapies including adaptive immune checkpoint inhibitors (ICIs) and chimeric antigen receptor (CAR) T cells, have developed the treatment of cancer in clinic, and most of them focus on activating T cell immunity. Although these strategies have obtained unprecedented clinical responses, only limited subsets of cancer patients could receive long-term benefits, highlighting the demand for identifying novel targets for the new era of tumor immunotherapy. Innate immunity has been demonstrated to play a determinative role in the tumor microenvironment (TME) and influence the clinical outcomes of tumor patients. A thorough comprehension of the innate immune cells that infiltrate tumors would allow for the development of new therapeutics. In this review, we outline the role and mechanism of innate immunity in TME. Moreover, we discuss innate immunity-based cancer immunotherapy in basic and clinical studies. Finally, we summarize the challenges in sufficiently motivating innate immune responses and the corresponding strategies and measures to improve anti-tumor efficacy. This review could aid the comprehension of innate immunity and inspire the creation of brand-new immunotherapies for the treatment of cancer.

## 1. Introduction

Cancer is one of the main leading causes of disease-associated death and it often results from genotoxic and non-genotoxic carcinogens [1]. Surgery, chemotherapy, radiotherapy and tumor immunotherapy are the principle treatment for tumors. Surgery is the treatment of choice for most solid tumors, but cannot be used for hematologic tumors and metastases and subclinical metastases of solid tumors. Compared to surgical treatment, chemotherapy is a systemic treatment for primary, metastatic and subclinical metastases of solid tumors as well as hematologic tumors. However, the poor selectivity of chemotherapeutic agents limits its clinical application. Radiation therapy protects tissue that is not affected by the tumor, produces less damage and does not require hospitalization, but the application of radiotherapy depends on the type, size and location of tumors. Compared with these traditional therapies, tumor immunotherapy can kill tumor cells by activating the host’s immune system [2,3]. The discipline of oncology has been undergoing an unprecedented revolution due to the massive advancement of immunotherapy in the treatment of cancer [4]. Infiltrating into the TME, immune cells assist in tuning the development of tumors [5,6]. In adaptive immunity, T cells participate in immune responses that are cell-mediated immune responses [7,8,9]. The treatment of solid tumors has been revolutionized by ICIs targeting PD-1/PD-L1. Blocking the PD-1/PD-L1 axis could enhance T cell infiltration and revitalize exhausted cytotoxic T cells, presenting impressive therapeutic efficacy in various cancers [10]. Interestingly, PD-L1 expression and sensitivity to immunotherapy of tumor cells are subject to metabolic reprogramming. For example, mitochondrial oxidative phosphorylation (OXPHOS) can regulate PD-L1 levels. Tumor-selective OXPHOS suppression nanoparticles have been reported to reactivate immunotherapy and may open a new therapeutic window for patients [11]. Furthermore, CAR T cells have shown impressive efficacy in the treatment of hematologic malignancies [12,13,14]. As the mechanisms of tumor development and treatment are further explored, many new strategies have also emerged. For instance, oxidative stress and reactive oxygen species (ROS) contribute to the development of cancer growth [15]. Myrrh, a traditional remedy, could be a possible therapeutic agent for cancer because of the powerful antioxidant activity of its extracts [15]. Pyroptosis is a form of programmed cell death, which is associated with tumor genesis, and therapy response. Inflammasomes are multimolecular complexes containing pattern recognition receptors that can recruit the adaptor protein containing apoptosis-associated speck-like protein containing a caspase recruitment domain and activate caspase-1. To date, six members of the gasdermin (GSDM) family have been identified, namely GSDMA, GSDMB, GSDMC, GSDMD, GSDME (DFNA5) and DFNB59. Inflammasomes can activate caspase-1 that can cleave GSDMD to induce pyroptosis [16]. It is reported that the virus-like particle can trigger the formation of an AIM2 inflammasome that can induce GSDMD-mediated pyroptosis, thus enhancing antitumor immunity [17]. The primary goal of the currently approved immunotherapies is to active T cell immunity, but innate immunity also has significant anti-tumor potential. The desire to identify novel targets in innate immunity has emerged in the new era of immunotherapy.

In the innate immune system, myeloid-derived suppressor cells (MDSCs), macrophages, neutrophils, natural killer (NK) cells, dendritic cells (DCs), mast cells (MCs) and helper innate lymphoid cells (ILCs) have important regulatory effects on tumor progression [18]. Some innate immune cells have the capacity for detecting and eliminating tumor cells through various mechanisms, such as intrinsic cytotoxicity of NK cells and macrophage phagocytosis. The interaction of the innate and adaptive immunity is exemplified by the uptake of tumor antigens by antigen-presenting cells (APCs), which results in cross-presentation and the priming of CD8^+^ T lymphocytes [19]. Innate immune cells are also involved in effector responses through antibody-dependent cellular phagocytosis (ADCP) and antibody-dependent cell cytotoxicity (ADCC) [20]. In this review, we state the roles of innate immunity in tumors, emphasizing the regulatory mechanisms by which innate immune cells recognize and respond to tumor cells and promote adaptive immune responses. We then highlight promising preclinical and clinical studies involving immunotherapies based on innate immune cells. Finally, we conclude the challenges in innate immunotherapy and propose corresponding strategies to improve the efficacy and facilitate its application in clinic.

## 2. Innate Immunity in TME

**Figure 1:**
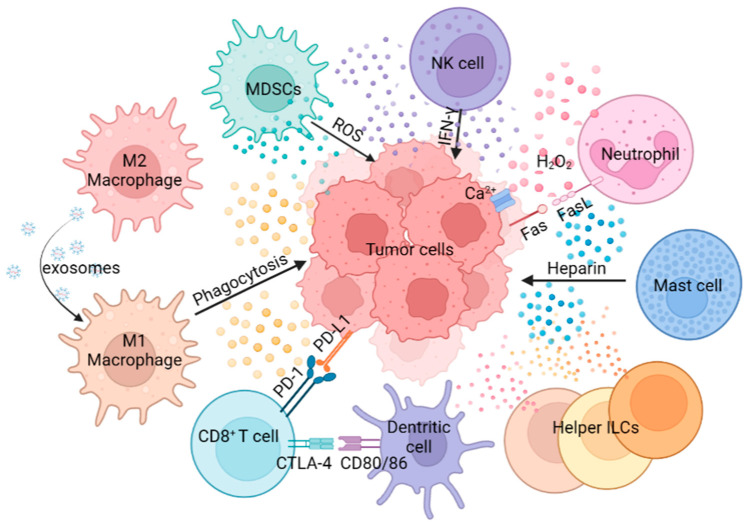
The role of innate immune cells in tumor progression. Innate immune cells show multiple roles in tumor progression through antigen presentation, phagocytosis, secretion of cytokines, direct killing effect, etc.

Composed of MDSCs, macrophages, neutrophils, NK cells, DCs, MCs and helper ILCs, the innate immunity plays a crucial role in defense against tumors (Figure 1). For example, macrophages can activate the tumor-killing activity of T cells through antigen cross-presentation. Antigen cross-presentation by macrophages can be achieved through the cytosolic and vacuolar pathways. In the cytosolic pathway, proteins are transported to the cell membrane, where they are degraded by the proteasome. Subsequently, the derived peptides are transferred via the transporter associated with antigen presentation to the endoplasmic reticulum (ER), where they are processed by aminopeptidases of the ER, or brought back to the antigen-containing endosome to be processed by insulin-regulated aminopeptidases. In this process, the peptide is loaded on the MHC-I. The other major cross-presentation pathway is the vacuolar pathway, in which proteins are processed by endosomal/lysosomal proteases and loaded on MHC-I within the endosomal/lysosomal compartments. The resulting peptide MHC-I molecular complex is recognized by CD8^+^ T cells expressing specific T cell receptors (TCRs). The signal from TCR recognition of antigen is transduced into CD8^+^ T cells, which triggers cytotoxic effects [21]. Neutrophils can promote tumor proliferation and invasion by secreting proteases, and can also inhibit tumor progression by secreting H_2_O_2_, based on the type, stage and location of the tumors [22]. NK cells achieve anti-tumor effects by mediating ADCC, and MCs can promote tumors or fight tumors by releasing cytokines, chemokines, eicosanoids, proteoglycans and biogenic amines [23,24]. A comprehensive understanding of innate immune cell function in the TME is vital for the design of effective cancer immunotherapy.

### 2.1. MDSCs

As a heterogeneous population of immature myeloid cells that possess potent immunosuppressive properties, MDSCs are typically classified into two major subsets based on their phenotypic, morphological and functional properties: mononuclear phagocyte-like MDSCs (M-MDSCs) and granulocyte-like MDSCs (G-MDSCs) [25,26,27]. G-MDSCs are predominantly abundant within the TME, whereas M-MDSCs primarily accumulate in the peripheral blood [28]. MDSCs play a crucial role in modulating immune responses and their dysregulation is associated with numerous diseases, including cancer, chronic infections, autoimmune disorders and inflammatory conditions [27]. In cancer, MDSCs suppress antitumor immune responses, promote tumor progression and contribute to immune evasion through a number of mechanisms, including boosting the production of Arg1, iNOS, ROS and nitrogen compounds like peroxynitrite (PNT) [29]. When Arg-1 is increased, L-arginine, an essential amino acid for T cell proliferation, could be metabolized into urea and L-ornithine [30]. When L-arginine is restricted, the up-regulation of iNOS contributes to NO production, which reacts with superoxide to produce PNT [31]. Subsequently, PNT causes the TCR nitration and nitrosylation, inhibiting the function of CD8^+^ T cells and promoting T cell tolerance [32,33]. Therefore, one crucial way of targeting MDSCs to reverse the immunosuppressive TME is to understand the basic mechanisms of MDSC recruitment into the TME [28].

### 2.2. Macrophages

Macrophages, which originate from myeloid-derived progenitor cells in the bone marrow, are the main phagocytes in tumors [34]. Macrophages can eliminate cellular debris clear pathogens and regulate inflammatory responses, which are essential for homeostasis maintenance [35]. Macrophages can be polarized into M1 phenotype and M2 phenotype [36]. M1 macrophages are characterized by producing pro-inflammatory cytokines such as interleukin (IL)-6, IL-12, and interferon (IFN)-γ, which possess anti-tumor properties. In addition, M1 macrophages recruit T helper type 1 (Th1) cells by secreting the chemokines CXCL9 and CXCL10, and promote T cell responses by upregulating the gene of antigen presentation and costimulatory molecules [35]. In contrast, M2 macrophages exhibit pro-tumor activities and produce immunosuppressive cytokines such as IL-4, IL-10, and transforming growth factor-*β* (TGF-*β*). In the TME, tumor-infiltrating macrophages, also known as tumor-associated macrophages (TAM), mainly exhibit an M2-like phenotype [37,38].

### 2.3. Neutrophils

Neutrophils, derived from the early committed neutrophil progenitor cells, are considered to be the most massive innate immune cells in the bone marrow and peripheral blood. These cells can respond quickly to inflammation, infection and injury. They can promote cancer progression through various mechanisms, such as angiogenesis, immunosuppression and cancer metastasis. In promoting tumor invasion, neutrophils secrete proteases that degrade structural proteins in the extracellular environment [39,40,41]. For instance, studies on breast cancer have shown that neutrophils can induce MMP-12 and MMP-13 to promote cancer metastasis [42]. Independent clinical studies have also confirmed the important role of neutrophils in tumor metastasis [43,44,45,46,47]. In addition, IL-1β promotes IL-17 production from γδT cells, leading to polarization of neutrophils in mice with mammary tumors [48]. Nonetheless, neutrophils can also exert anti-tumor by, for example, secreting H_2_O_2_ to cause an influx of Ca^2+^ and inducing apoptosis in tumor cells via Fas ligand/Fas interaction [22].

### 2.4. NK Cells

NK cells are cytotoxic lymphocytes that hold the capacity to kill cancerous cells. In general, NK cells can kill tumor cells via direct cytotoxicity and pro-inflammatory cytokines production [24]. “Missing-self” is one mechanism of direct cytotoxicity. Immunoreceptor tyrosine-based inhibitory motifs (ITIMs) can engage with the major histocompatibility complex-I (MHC-I) to stop NK cells from destroying ordinary cells, which means NK cell activation can be inhibited by the binding of MHC-I. However, tumor cells always lack or only very faintly express MHC-I in order to escape CD8^+^ T cell-mediated cytotoxicity. Thus, NK cells can recognize and reply to this missing-self phenotype [49]. ADCC is another crucial mechanism of NK cell-mediated direct cytotoxicity [24]. Additionally, NK cells express significant quantities of IFN-γ and TNF. They can not only enhance cytotoxic CD8^+^ T cell response, but also can they suppress the proliferation and angiogenesis of tumor cells while they promote the apoptosis of cancerous cells [50].

### 2.5. DCs

First discovered in 1973 by Ralph Steinman, DC is one of the most important APCs, which plays a significant role in launching and modulating innate and adaptive immune responses [51]. DCs comprise four main subsets: cDC1s, cDC2s, plasmacytoid DCs (pDCs) and monocyte-derived DCs (MoDCs). In the TME, DCs recognize, uptake, process and present tumor-associated antigens (TAAs) to T cells to shape T cell responses [52]. Distinct DCs have different T cell priming abilities. cDC1s excel at cross-presentation antigens, which could activate CD8^+^ T cells and promote Th1 cell polarization of CD4^+^ T cells. cDC2s are essential to inducing CD4^+^ T cell responses [53]. pDCs could effectively prime CD8^+^ T cells while showing poor priming of naive T cells [54,55,56]. MoDCs are mainly generated in inflammatory responses and are critical to the differentiation of CD4^+^ T cells. When perceiving appropriate cues, DCs express costimulatory molecules such as CD80, CD86, CD137L, OX40L, GITRL, CD70 and CD40 to regulate DC-mediated T cell priming to influence T cell-mediated immunotherapy in TME [51]. DCs also upregulated chemokine receptors such as cc-chemokine receptor 7 (CCR7) [57]. Studies show that CCR7 is critical for the migration of tumor-infiltrating DCs and can impact DCs recruitment into the TME. Chemokines such as CXC-chemokine ligand 9 (CXCL9) and CXCL10 are also produced by DCs to facilitate the recruitment of CD8^+^ T cells in the TME [58]. In addition, DCs could produce cytokines such as IL-12 and type 1 TNF; IL-12s are pivotal to the initiation of Th1 cells and CD8^+^ T cells, and type 1 TNFs have been used to treat patients with cancer [19,59,60,61].

### 2.6. MCs

MCs are derived from bone marrow precursors, which exert either pro- or anti-tumor effects by secreting cytokines, chemokines, eicosanoids, proteoglycans and biogenic amines after activation [23]. Studies demonstrate that IL-33-mediated MC activation promotes TAM infiltration and tumorigenesis by secreting CSF2, CCL3 and IL-6 in gastric cancer [62]. Eicosanoids, such as LTC4, LTB4 and PGD2, are also secreted upon MC activation. Transforming LTC4 to LTD4 by γ-Glutamyl transpeptidase 1 (GGT1) and GGT5, enzymes that hydrolyze γ-glutamyl of glutathione, results in lung cancer cell migration and survival via CysLT1 [63,64]. LTB4 has the potential to be a biomarker for predicting the effect of bestatin in colorectal cancer (CRC) [65]. PGD2 can suppress tumorigenesis and metastasis via PGD2/PTGDR2 signaling [66,67]. Mast cell-derived heparin is a kind of proteoglycan that could increase angiogenesis. In mouse EL-4 lymphoma and MC-38 colon cancer, histamine produced by MCs could target MDSCs and enhance the anti-tumor efficacy of checkpoint blocking [68].

### 2.7. Helper ILCs

ILCs are a subset of lymphocytes that lack antigen-specific receptors. There are five subsets of ILCs: NK cells, lymphoid tissue inducer cells, and groups 1, 2, and 3 (ILC1, ILC2, and ILC3) [69,70]. The Helper ILC family, which consists of ILC1, ILC2, and ILC3, can be utilized to induce and stimulate anti-tumor responses [71]. ILC1s have the capacity to express IFN-γ, NKp46, and T-bet [72]. Type 2 cytokines, including IL-5 and IL-13, are produced by ILC2s in response to TSLP, IL-18, IL-33, or IL-25 [73]. Tumor-infiltrating helper ILCs can either stimulate or restrict the proliferation of cancer cells through various mechanisms. ILC1s can produce IFN-γ and granzymes to suppress tumor growth, but express immune checkpoints on their surface may impair their function and promote tumor growth [72]. Upon stimulation with IL-33, ILC2s can secrete high levels of IL-5, GM-CSF, and CCL5, which can activate and recruit anti-tumorigenic eosinophils, DCs, and CD8^+^ T cells [74]. In contrast, the production of immune checkpoint molecules like PD-1 and the cytokines IL-4 and IL-13 dampens ILC2 activity and accelerates tumor growth [75]. Under the regulation of TGF-*β* signaling, ILC3s can also convert to ILC1s or regulatory ILCs [76]. According to the type of tumor, ILC3s produce cytokines including IL-17 and IL-22 that promote or inhibit tumor development [77,78].

## 3. Immunotherapies Targeting Innate Immune System

**Figure 2:**
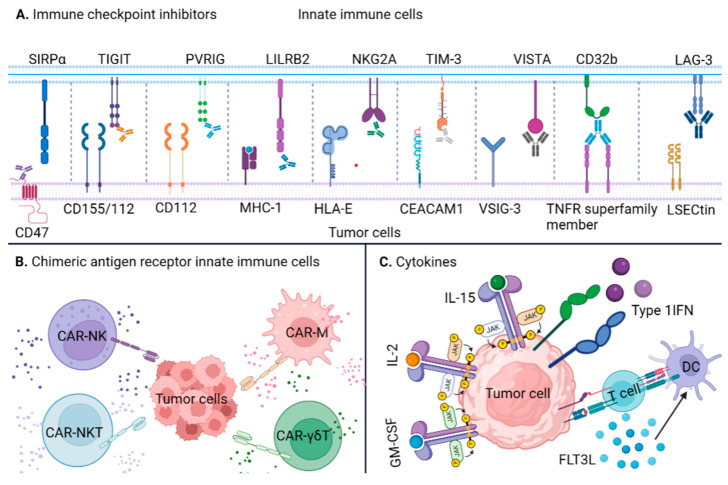
Innate immune cell-based immunotherapy. Innate immunotherapies include innate ICIs, CAR innate immune cells and cytokines. (**A**) innate immunity-based ICIs; (**B**) CAR innate immune cells; (**C**) cytokines in innate immunity.

Current immunotherapies including ICIs, cell therapies and tumor vaccines, present potent antitumor efficiency [79,80,81]. Through disrupting the immune checkpoint pathway, ICIs have become the most widely used agents in cancer immunotherapy [82]. CAR T cell therapy was developed from adoptive T cell transfer, which typically modifies and redirects patients’ own T cells to recognize and kill tumor cells. Recently, CAR-T cell therapy has achieved notable success in the treatment of hematologic malignancies [79]. Through inducing APC cell-mediated immune responses, tumor vaccines could be designed to eradicate malignant cells [83]. Recently, immunotherapies based on innate immune cells have emerged, including innate ICIs, CAR innate immune cells and cytokine therapy. Innate ICIs achieve anti-tumor purposes by targeting checkpoints such as SIRPα, TIGIT, PVRIG, LILRB2, NKG2A, LAG-3, TIM-3, VISTA and CD32B (Table 1; Figure 2A) [84]. NK cells, NKT cells, γδT cells and macrophages engineered with CAR may be redirected effectively towards specific antigens (Table 1; Figure 2B) [85]. GM-CSF, type I IFNs, FLT3L, IL-2 and IL-15 can promote the development and proliferation of APCs as well as the activation of NK cells (Table 2; Figure 2C) [86].

### 3.1. Innate ICIs

Innate ICIs have the potential to both provide stimulation and disrupt inhibitory relationships between malignancies and phagocytes or NK cells. These checkpoints include Signal-regulatory protein-α (SIRPα), T-cell Ig and ITIM domain (TIGIT), Poliovirus receptor-related Ig domain containing protein(PVRIG), Leukocyte immunoglobulin-like receptor B (LILRB2), NK group 2 member A (NKG2A), T cell immunoglobulin and mucin domain 3 (TIM-3), V-domain immunoglobulin suppressor of T cell activation (VISTA), FcγRIIB (CD32B), lymphocyte activation gene 3 protein (LAG-3) and cytotoxic T-lymphocyte-associated protein 4 (CTLA-4) [84,86].

The ligand of SIRPα is CD47, which is abundantly presented on various cancerous cells [87]. The SIRPα/CD47 interaction leads to phosphorylating the ITIMs and disrupting the cytoskeleton, and then inhibiting phagocytosis [87]. Blocking the SIRPα/CD47 axis could not only activate adaptive anti-tumor immune responses by promoting antigen cross-presentation by APCs, but also could activate innate immune responses by enhancing the cancer cell clearance by macrophages and DCs [84,88]. TTI-621 is a fusion protein consisting of the CD47 binding domain of human SIRPα and linked to the Fc region of IgG1. Studies show that TTI-621 could block the “do not eat me” signal of CD47 and boost phagocytosis and anti-tumor effect, and intra-tumoral injections of TTI-621 are used to treat patients with mycosis fungoides and/or Sézary syndrome, which result in a rapid decrease in tumor size [89,90,91].

TIGIT is a suppressible receptor presented on NK cells, CD4^+^ T cells, CD8^+^ T cells and regulatory T cells [86]. In addition, the ligands of TIGIT, CD112 and CD155, are presented on tumor cells [86]. According to preclinical research, TIGIT blockade could enhance NK cell activity and CD8^+^ T cell cytotoxicity [92,93,94,95]. Many monoclonal antibodies (mAbs) have been designed to prevent the inhibitory function of human TIGIT. Tiragolumab is a humanized anti-TIGIT IgG1 mAb created to prevent TIGIT from interacting with CD155. Tiragoulumab alone was used to treat 24 cancer patients who were refractory in an Ia/Ib phase experiment, and the medication resulted in stable disease. Additionally, 49 individuals received treatment with tiragolumab plus atezolizumab, which led to improvements in 3 patients, including partial responses in NSCLC and head and neck squamous cell carcinoma (HNSCC) [84]. Similar to TIGIT, PVRIG, presented on NK cells and CD8^+^ T cells, is a suppressible receptor that could identify CD112 on tumor cells [96]. Preclinical studies suggest that anti-PVRIG therapy could enhance CD8^+^ T cell cytotoxicity and NK cell anti-tumor effect [94,97].

LILRB2 is presented on innate immune cells and CD4^+^ T cells, which could interact with MHC-I on nucleated cells [98]. Acetylated by acetyltransferase CREB-binding protein (CBP), STAT6 could suppress M2 polarization during macrophage maturation [99]. In the presence of macrophage colony-stimulating factor M-CSF and IL-4, LILRB2 inhibitors can regulate M2 polarization by inhibiting STAT6 activation [100]. LILRB2 antagonism also could improve NF-κB and STAT1 activation and reduce the suppressive effect of macrophages on T cell proliferation. Additionally, when combined with anti-PD-L1, LILRB2 blockade could remodel the TME and provoke anti-tumor immunity [100].

NKG2A and CD94 form a suppressible receptor which is presented on NK cells and CD8^+^ T cells and is capable of recognizing HLA-E [86]. Both hematologic and solid tumors upregulate HLA-E expression, which has been linked to a worse prognosis and lower NK cell cytotoxicity [92,101]. In cancer patients, combining NKG2A inhibition with PD-L1 blockade may increase the anti-tumor efficacy of NK and CD8^+^ T cells. Monalizumab is an anti-NKG2A antibody that, when combined with PD-x axis blockade, can improve NK cell activity against multiple tumor cells and promote the CD8^+^ T cell effect. Monalizumab and cetuximab were used in a trial to treat patients with HNSCC, and led to a 31% objective response rate [102].

While immune checkpoints such as LAG-3, TIM-3, VISTA and CTLA-4 are primarily adaptive immunological checkpoints, they may also have innate immune effects. It has been shown that LAG-3 reduces the effector functions of NK cells, NKT cells and DCs [86]. Through inhibiting tumor nucleic acid sensing, TIM-3 suppresses tumor detection of DCs [103]. Elevated expression of VISTA was reported in macrophages, DCs and monocytes [104]. CTLA-4 binds to its ligands, CD86 and CD80 that are expressed on APCs such as DCs and macrophages, leading to the depletion of naive and memory T cells [105]. Many areas of current and future research will probably elucidate the significance of these checkpoints in tumor treatment.

### 3.2. Innate Immune Cells Engineered with CARs

Consisting of an extracellular region, hinge region, transmembrane domain and intracellular domain, CAR is a functional chimeric antigen receptor that typically confers an immune cell with the specificity for TAAs [106]. Usually, single-chain variable fragment in the extracellular region can specifically recognize antigens expressed on tumor cells. The intracellular domain usually includes co-stimulatory molecules and the CD3ζ domain of TCR. When the CAR recognizes the specific antigen, immunoreceptor tyrosine-based activation motifs are phosphorylated, thereby recruiting adaptor molecules, regulating downstream pathways and activating immune cells [107]. Innate immune cells such as macrophages, NK cells and NKT cells can also be used for CAR redirection despite the fact that T cells predominate in CAR-based immunotherapy [85].

CAR-M treatment is based on a 1 week manufacturing procedure that begins with a patient’s own blood. Subcutaneous G-CSF treatment mobilizes monocytes prior to leukapheresis and CD14^+^ monocyte selection. Following ex vivo differentiation of monocytes into macrophages, Ad5f35—which encodes the CAR transgene—is next introduced into the cells. Surprisingly, CAR-M, generated with Ad5f35, could eradicate tumor cells more efficiently than M1 macrophages. Additionally, Ad5f35 transduced macrophages did not differentiate into M2 macrophages when stimulated with IL-4, IL-10, IL-13 or a tumor-conditioned medium. Notably, CAR-M macrophages could upregulate antigen presentation pathways and exhibit increased T cell activation capability as compared to control macrophages [108].

The initial signaling domain of CAR-NK cells is frequently CD3, and the costimulatory domain is either CD28 or CD137. Due to their improved tumor-specific targeting and cytotoxicity, CAR NK cells have been employed to identify cancer cells [109]. NKT cells are recognized by the production of TCRs and NK cell lineage markers [110]. Studies have shown that NKT modified with ganglioside GD2-specific CARs has a killing effect on GD2-positive tumor cells as well as CD1d-positive M2 macrophages in vitro, and CD19-specific CAR-modified NKT cells can be used to treat B-cell lymphoma [111,112]. CAR γδT cells can recognize multiple antigens and are tumor cytotoxic [113]. CD19-directed CAR γδT cells target both CD19-positive cell lines and CD19-negative leukemia cells [114].

### 3.3. Cytokines

Innate immunity plays a central role in promoting T cell effector functions. To enhance this effect, many attempts have been made and the results show that cytokines, such as GM-CSF, type I IFNs, FLT3L, IL-2 and IL-15, can activate NK cells or promote the maturation and multiplication of APC [86].

GM-CSF could recruit NK cells and DCs to promote the presentation of TAAs thereby mediating anti-tumor effects. In mice, a GM-CSF injection could stimulate immunity and present long-lasting anti-tumor responses [86]. In patients with melanoma, an intra-tumoral injection of GM-CSF could contribute to increasing the number of tumor-infiltrating DCs. Talimogene laherparepvec (T-VEC) is an engineered oncolytic virus with the gene that encodes GM-CSF, and it is demonstrated that its efficacy is superior to GM-CSF in patients with advanced melanoma [115]. Studies suggest that the therapeutic effect of T-VEC is related to the increase in tumor-specific T cells and the decrease in T_reg_ cells [115].

As a protein localized on the ER membrane, the stimulator of interferon genes (STING) plays a vital role in activating immune responses [116,117]. Cyclic GMP-AMP synthase (cGAS) is a cellular DNA sensor that could recognize pathogen-released cytoplasmic double-stranded DNA (dsDNA). Cytosolic dsDNA, originating from various sources such as chromosomal instability, acute genomic stressors and mitochondrial dysfunction, is frequently abundant in cancer cells [118]. cGAS binds to its DNA ligand and forms a dimer, in which the DNA is sandwiched between two cGAS protomers. Active cGAS binds to ATP and GTP and catalyzes the condensation reaction of these two nucleotides to produce 2′,3′-cyclic GMP–AMP (2′,3′-cGAMP). Then, cGAMP binds to STING, and results in TANK-binding kinase 1-dependent phosphorylation of interferon regulatory factor 3 (IRF3). Upon activation, IRF3 translocates into the nucleus where it initiates the transcriptional activation of type I IFNs. Additionally, the cGAS-STING signaling pathway induces the expression of genes encoding pro-inflammatory cytokines via NF-κB activation [118,119]. Type I IFNs are significant for the spontaneous activation of tumor-specific CD8^+^ T cells and have considerable influence on the capacity of APCs to deliver dead cell-associated antigens [120]. Moreover, type 1 INF could promote the generation of CXCL9 and CXCL10 to impact the activity and recruitment of NK cells in the TME [121]. Of note, interferon-induced tetratricopeptide repeat (IFIT) proteins could influence the translation of a diverse array of viruses. As one of the most crucial members of both the IFIT family and the interferon-stimulated gene family, IFIT3 is vital for cell proliferation, differentiation and cancer metastasis, eliciting an important role in antiviral innate immunity [122]. A study shows that the addition of IFN2a can improve the anti-tumor ability and survival of mice when using denileukin diftitox to treat ovarian cancer [123]. In preclinical trials, using antibody-cytokine conjugates to send type 1 INF to the TME has resulted in an anti-tumor effect with no obvious toxicity [124,125,126].

FLT3L is a kind of cytokine manufactured by NK cells, which can effectively enhance DCs’ survival and proliferation [127]. A study shows that injecting FLT3L into mice can increase the number of CD8^+^ T cells and the ratio of CD8^+^ T cells to T_reg_ cells in the TME, which result in the proliferation of cross-presenting DC and enhancement of the responses to intra-tumoral TLR3 agonists [128]. In a current trial, patients with advanced stage NHL were treated with the combination of FLT3L, a TLR3 agonist and localized radiotherapy, which has yielded an encouraging result [129].

Both IL-2 and IL-15 could bind with a CD122-CD132 receptor, thus treatment with IL-2 or IL-15 can induce the activation and extension of NK cells, CD8^+^ T cells, NKT cells and γδT cells [86]. However, IL-2 also has a high affinity to CD25-CD122-CD132 complex that is also presented on T_reg_ cells. Thus, the reason why treatment with IL-2 results in a significant decrease in tumor in mouse models but yields a poor result in some patients with melanoma or renal cell cancer is that IL-2 promotes the proliferation of T_reg_ cells [86,130]. IL-15 does not bind with CD25 or induce the expansion of the T_reg_ cell population and therefore, shows better anti-tumor activity and lower toxicity than IL-2.

## 4. Challenges and Future Directions of Innate Immunotherapy

Although innate immunotherapy has significantly improved the treatment of tumors by using the body’s own defense mechanisms to successfully combat a variety of cancers, there are immune-related side effects that can impact several organ systems.

ICI therapies focus on common anti-tumor mechanisms to provide generalizability to a variety of cancers. The cardiovascular system is just one of the major organ systems that can experience ICI-related adverse effects. Studies show that ICI-related cardiotoxicity includes myocarditis, atherosclerosis and arrhythmias. Among them, the most well-known and most likely fatal is myocarditis, with a mortality rate of nearly 50% [131]. These side effects could be reduced with supportive care and immunosuppression with corticosteroids. While SIRP/CD47 inhibition causes the phagocytosis of tumors, red blood cells (RBC) are also prone to phagocytosis that is dose-dependent and may result in anemia and complications due to infusions [132]. When the Fc region of the antibody binds to the Fc receptor (FcγR) on macrophages, an antibody-dependent cellular phagocytosis (ADCP) is initiated. As noted above, blockade of CD47/SIRPα, the “don’t eat me” axis, promotes phagocytosis of tumor cells. CD47 is also widely expressed in normal human cells, especially on RBC, which leads to macrophages phagocytosis of RBC via ADCP, leading to anemia. Therefore, using Fc that cannot bind to FcγR or have a weak binding effect (such as IgG2 and IgG4) is an effective strategy to reduce anemia side effects. In addition, adjusting the dose of the drug to priming dosing and reducing the affinity of the antibody to some extent may also reduce the risk of anemia [84].

Adoptive new treatment with CAR immunotherapy has made significant progress. CAR immunotherapy for solid tumors, however, is far behind. Manufacturing of cells, a dearth of tumor-specific antigens and ineffective penetration into tumor locations are the principal obstacles to CAR immunotherapy in solid tumors [109]. Although the benefits of CAR NK cell treatment are clear, there are also substantial drawbacks. The current state of CAR NK manufacturing and storage, optimization of CAR NK structure, maintaining cytotoxic effects at the immunosuppressive TME and minimization of off-target toxicity are all areas of active research. Additionally, the half-life of NK cells is less than 10 days, which limits the off-target toxicity, but it may also represent a limitation in persistence, function and the durability of the response, meaning multiple administrations could be necessary to obtain a long-lasting reaction [133]. Currently, researchers are working to enhance the persistence of CAR NK cells in vivo. MyD88/CD40 are signaling molecules for NK cell activation and proliferation. In one study, inducible MyD88/CD40 (iMC) CAR NK cells were extended to CAR NK cells and enhanced the tumor killing efficacy of CAR-NK by increasing cytotoxic function, cytokine secretion and proliferation. The cytotoxicity and persistence of NK cells could also be tuned by IL-15. Autocrine IL-15 coupled with iMC could further drive CAR-NK cell proliferation and survival in vivo, providing a novel strategy to effectively improve the persistence of CAR NK cells [134]. Only one clinical trial for CAR macrophage has been started, and there have been no findings announced as yet. CAR M enables it to target tumor cells expressing specific antigens. The recognition of tumor antigens can induce several macrophage-mediated anticancer mechanisms: (1) triggering macrophage-mediated ADCC to kill tumor cells; (2) upon binding to specific tumor antigens, CAR receptors activate intracellular signaling pathways, induce phagocytosis and rapidly present tumor antigens to activate T cell-mediated immunity; (3) inhibiting pro-tumor polarization of macrophages and activating pro-inflammatory response pathways; (4) secreting pro-inflammatory cytokines such as interferon-gamma to recruit and activate other immune cells; (5) CAR macrophages continuously kill cancer cells, and they may also stimulate adaptive immune response, thus providing persistent anti-tumor immunity; (6) increasing the expression of MHC-I and MHC-II, thus enhancing T-cell activation; and (7) promoting the intratumoral infiltration of CD4^+^ and CD8^+^ T cells, NK cells and DCs, thereby activating a systemic immune response against solid tumors [135]. In TME of solid tumors, there are usually few T cells but abundant macrophages. This may make CAR M more efficient than CAR T and CAR NK when encountering a “cold” tumor, but still needs a long time before widespread clinical use [109].

Immunostimulatory cytokines are important to immune system regulation and exhibit significant promise as immunotherapeutic drugs. However, substantial toxicities brought by systemic immune activation have restricted their clinical usage. Fusions of cytokines with mAbs or antibody fragments have been designed in order to increase efficacy and decrease systemic toxicity [136,137]. Additionally, cytokine fusions with inhibitory moieties that can be broken down by tumor-associated proteases, lowering the cytokine’s affinity to improve targeting, and variations of oligomeric cytokines that are initiated by colocalization of monomeric subunits can all be used to ensure that cytokines are only activated at tumor sites [138,139,140]. A conditionally active cytokine system has been developed for monomeric cytokines such as IL-2 to prevent the persistent off-target activity that is typically seen with systemic cytokine and immune-cytokine treatment [141]. There is controversy around the usage of GM-CSF as an immunoadjuvant because studies suggest that using it as an adjuvant could result in immunosuppression rather than immune stimulation [70,142,143]. Even though GM-CSF has a long history and a large body of clinical evidence demonstrating its safety and effectiveness as an immune adjuvant, it is important to carefully evaluate the studies previously mentioned and take into account the potential mechanisms of GM-CSF-induced immunosuppression as suggested above.

## 5. Conclusions

In this review, we discussed in depth the roles and mechanisms of innate immunity in the TME and immunotherapies targeting the innate immune system, which include innate ICIs, innate immune cells engineered with CAR and cytokines. Then we discussed the challenges and future strategies of innate immunotherapy. Although innate immunity brings new opportunities for oncology treatment, there are still challenges such as inadequate eradication rate, toxic side effects and the potential need for repeated dosing, which means we still need to further investigate how to adequately use these innate immune cells in cancer immunotherapy. From multiple perspectives, multi-strategy combination therapy is the way forward for the treatment of tumors.

## Figures and Tables

**Table 1:** Selected clinical trials of innate immunity-based ICIs and engineered cellular immunotherapies.

Target	NCT Number	Status	Phase	Cancer Type	Interventions	Enrollment
SIRPα	NCT05076591	Recruiting	I	Advanced Breast Cancer, Advanced Gastric Cancer	IMM2902	135
	NCT05276310	Recruiting	I	Advanced Cancer	IMC-002	24
	NCT05192512	Recruiting	I	Advanced Cancer	TQB2928	180
	NCT05507541	Not yet recruiting	II	Multiple cancer types	TTI-622 and TTI-621 in combination with Pembrolizumab	62
	NCT05263271	Recruiting	I	AML; MDS	Gentulizumab	58
TIGIT&PVRIG	NCT04570839	Recruiting	I/II	Endometrial Neoplasms, Ovarian Cancer, HNC	COM701 in combination with BMS-986207 and Nivolumab	100
	NCT03667716	Recruiting	I	Ovarian Cancer, Breast Cancer	COM701 and Nivolumab	140
LILRB2	NCT04669899	Recruiting	I/II	Multiple cancer types	JTX-8064	281
	NCT05054348	Recruiting	I	Solid Tumor	IO-108, Pembrolizumab	36
NKG2A	NCT05162755	Recruiting	I	Solid Tumor	S095029	129
	NCT04914351	Not yet recruiting	I	Solid Tumor	HY-0102	32
	NCT05414032	Not yet recruiting	II	Advanced HNSCC	Monalizumab, Cetuximab	200
LAG-3	NCT03252938	Recruiting	I	Peritoneal Carcinomatosis	IMP321	45
	NCT05101109	Recruiting	I	Advanced Solid Tumor	ABL501	36
	NCT05078593	Recruiting	I	Solid Tumor, Lymphoma	HLX26	11
	NCT04618393	Recruiting	I/II	Advanced Solid Tumor	EMB-02	43
	NCT05577182	Not yet recruiting	I	Advanced Malignancies	INCA32459-101	120
TIM-3	NCT05357651	Not yet recruiting	I	Solid Tumor, Lymphoma	LB1410	100
	NCT02817633	Recruiting	I	Multiple cancer types	TSR-022	369
	NCT04370704	Recruiting	I/II	Melanoma	INCAGN02385 in combination with INCAGN02390 and INCMGA00012	146
	NCT04931654	Recruiting	I/II	NSCLC	AZD7789	81
	NCT05367401	Not yet recruiting	I/II	MDS, AML	Sabatolimab, Magrolimab	63
VISTA	NCT04475523	Recruiting	I	Solid Tumor	CI-8993	50
	NCT05082610	Recruiting	I	NSCLC	HMBD-002, Pembrolizumab	240
CD32B	NCT04219254	Recruiting	I/II	Solid Tumor	BI-1206 in combination with Pembrolizumab	90
	NCT05555251	Recruiting	I/II	HER2-positive Breast Cancer, HER2-positive Gastric Cancer	BI-1607 in combination with Trastuzumab	116
	NCT03571568	Recruiting	I/II	Indolent B-Cell NHL	BI1206 in combination with Rituximab	30
CAR NK	NCT05213195	Recruiting	I	Refractory Metastatic CRC	NKG2D CAR-NK cells	38
	NCT05507593	Recruiting	I	SCLC	DLL3-CAR-NK cells	18
	NCT04662788	Not yet recruiting	I	Hematological Malignancies	NK cells/Combined Monoclonal Antibodies	36
	NCT05410717	Recruiting	I/II	Ovarian cancer, Testis cancer	Claudin6 targeting CAR-NK cells	40
CAR M	NCT04660929	Recruiting	I	Multiple cancer types	CT-0508	18
CAR NKT	NCT03294954	Recruiting	I	Neuroblastoma	GD2 Specific CAR and IL-15 Expressing Autologous NKT Cells	36
	NCT03774654	Recruiting	I	Multiple cancer types	CD19.CAR-aNKT cells	48
	NCT05487651	Not yet recruiting	I	NHL, BCL, DLBCL	KUR-502	36

AML: acute myelocytic leukemia; MDS: myelodysplastic syndrome; HNSCC: head and neck squamous cell carcinoma; HNC: head and neck cancer; CRC: colorectal adenocarcinoma; DLBCL: diffuse large B cell lymphomas; BCL: B cell lymphoma; HNSCLC: non-small-cell lung cancer; SCLC: small-cell lung cancer; NHL: non-hodgkin lymphoma.

**Table 2:** Selected clinical trials of cytokines in innate immunity.

Target	NCT Number	Status	Phase	Cancer Type	Interventions	Enrollment
GM-CSF	NCT05284214	Not yet recruiting	II	Solid Tumor	Sargramostim, Ipilimumab-containing therapy	65
	NCT05530200	Not yet recruiting	II	Metastatic Solid Tumor	PD-L1 inhibitor, GM-CSF, IL-2	56
	NCT03866525	Recruiting	I/II	Gastrointestinal Cancer	OH2	300
	NCT05292417	Recruiting	II	CRC	GM-CSF in combination with Sintilimab and Fruquintinib	71
	NCT04725331	Recruiting	I/II	Metastatic Cancer	BT-001, Pembrolizumab	48
type I IFNs	NCT04053673	Recruiting	I	Solid Tumor	RBN-2397	130
	NCT05127590	Recruiting	I/II	Advanced NSCLC	RBN-2397	50
	NCT04544007	Recruiting	II	Glioma	Poly ICLC	20
FLT3L	NCT05029999	Recruiting	I	Metastatic Triple Negative Breast Cancer	PLD Chemotherapy, CDX-1140, CDX-301	45
	NCT04491084	Recruiting	I/II	NSCLC	CDX-301, CDX-1140	46
	NCT03789097	Recruiting	I/II	NHL; HNSCC; Metastatic Breast Cancer	Pembrolizumab, CDX-301, Poly ICLC	56
	NCT04616248	Not yet recruiting	I	Multiple cancer types	CDX-301, CDX-1140, Poly ICLC	18
IL-2	NCT05307874	Recruiting	I/II	Solid Tumor	ICT01 in combination with IL-2	75
	NCT05267626	Recruiting	I/II	Advanced Solid Tumor	AU-007	69
	NCT04862767	Recruiting	I	Solid Tumor	TASO-001 in combination with IL-2	9
	NCT05493566	Not yet recruiting	I	Lung Cancer	IL-2 in combination with Pembrolizumab	15
	NCT05538624	Not yet recruiting	I/II	Multiple cancer types	AVB-001	44
IL-15	NCT04294576	Recruiting	I	Advanced/Metastatic Solid Tumor	BJ-001	92
	NCT05470283	Recruiting	I	Metastatic Melanoma	OBX-115	30
	NCT05445882	Not yet recruiting	II	Castration-Resistant PCA	N-803TET	28
	NCT05266612	Not yet recruiting	I	Solid Tumor	VG2025	12
	NCT05359211	Recruiting	I	Multiple cancer types	NKTR-255	24

CRC: colorectal adenocarcinoma; TET: thymic epithelial tumor; NSCLC: non-small-cell lung cancer; NHL: non-hodgkin lymphoma; HNSCC: head and neck squamous cell carcinoma; PCA: prostate cancer.

## Data Availability

No new data were created or analyzed in this study. Data sharing is not applicable to this article.
